# Bowel Ischemia: A Life‐Threatening Complication of an Infected Urachal Cyst in an Adolescent

**DOI:** 10.1002/ccr3.72598

**Published:** 2026-04-26

**Authors:** Sheeraz Ali, FNU Saliha, Bilal Ahmad, Abdul Eizad Asif, Khalil El Abdi, Bilal Aslam, Fazeela Bibi, Said Hamid Sadat

**Affiliations:** ^1^ Liaquat University of Medical and Health Sciences Jamshoro Pakistan; ^2^ The University of Lahore Lahore Pakistan; ^3^ Shalamar Medical and Dental College Lahore Pakistan; ^4^ Faculty of Medicine and Pharmacy of Rabat Mohammed V University Rabat Morocco; ^5^ Jinnah Medical and Dental College Karachi Pakistan; ^6^ Kabul University of Medical Sciences Abu Ali Ibn Sina Kabul Pakistan

**Keywords:** acute abdomen, bowel ischemia, intestinal resection, pediatric surgery, urachal cyst

## Abstract

While infection is a recognized complication of congenital urachal cysts, progression to an inflammatory phlegmon causing bowel ischemia is an exceptionally rare and life‐threatening event in the pediatric population. We report the case of a 14‐year‐old male with a 7‐year history of an infraumbilical mass who presented with an acute abdomen. Contrast‐enhanced computed tomography revealed a 7.1 cm urachal phlegmon with a central necrotic core and dense adherence to the adjacent ileum. Exploratory laparotomy confirmed irreversible segmental small bowel ischemia, necessitating en‐bloc excision of the urachal remnant and the affected 12 cm ileal segment with a primary anastomosis. Histopathology confirmed a benign infected cyst with associated transmural bowel necrosis. This case highlights that long‐standing urachal anomalies can evolve into aggressive inflammatory masses capable of precipitating visceral compromise. It underscores the importance of a high index of suspicion for rare complications and supports the consideration of definitive surgical excision for symptomatic remnants to mitigate the risk of severe morbidity.

## Key Clinical Message

Symptomatic urachal cysts can evolve into aggressive inflammatory masses. This case, which resulted in bowel ischemia, highlights the importance of advanced imaging for diagnosis and supports considering definitive surgical excision for symptomatic remnants to prevent rare but severe visceral complications.

## 1 Introduction

The urachus, an embryologic remnant of the allantois connecting the fetal bladder to the umbilicus, typically obliterates after birth to form the median umbilical ligament [1]. Failure of this process results in a spectrum of urachal anomalies, including patent urachus, sinus, diverticulum, and cyst, with the cystic form being the most common variant [2]. Urachal cysts are typically located in the extraperitoneal space and often remain clinically silent, but they can become symptomatic, most commonly due to secondary infection [1, 3, 4].

An infected urachal cyst often presents with nonspecific findings such as suprapubic pain, fever, and a palpable infraumbilical mass, creating a diagnostic challenge that can mimic more common conditions like appendicitis or Meckel's diverticulitis [5]. While chronic inflammation is known to result in the formation of adhesions to adjacent structures [6], the progression to a severe, phlegmonous mass capable of causing vascular compromise and segmental bowel ischemia is an exceptionally rare and poorly documented complication in the pediatric population.

Herein, we report the case of an adolescent male with a long‐standing infected urachal cyst that precipitated segmental small bowel ischemia requiring en‐bloc intestinal resection. This report aims to highlight the diagnostic and therapeutic challenges of this severe visceral complication and underscore the potential for symptomatic urachal anomalies to evolve into aggressive inflammatory processes.

## 2 Case History

A 14‐year‐old male presented to the emergency department with a 4‐day history of acute, severe hypogastric pain, high‐grade fever (38.7°C), and obstipation. His medical history was notable for a 7‐year chronic, infraumbilical mass with occasional, minor purulent discharge. On physical examination, the patient was acutely unwell. The abdomen was soft, with a firm, fixed, and exquisitely tender 8 cm infraumbilical mass. The overlying skin exhibited marked erythema and warmth, consistent with a phlegmon.

## 3 Investigations and Differential Diagnosis

**FIGURE 1:**
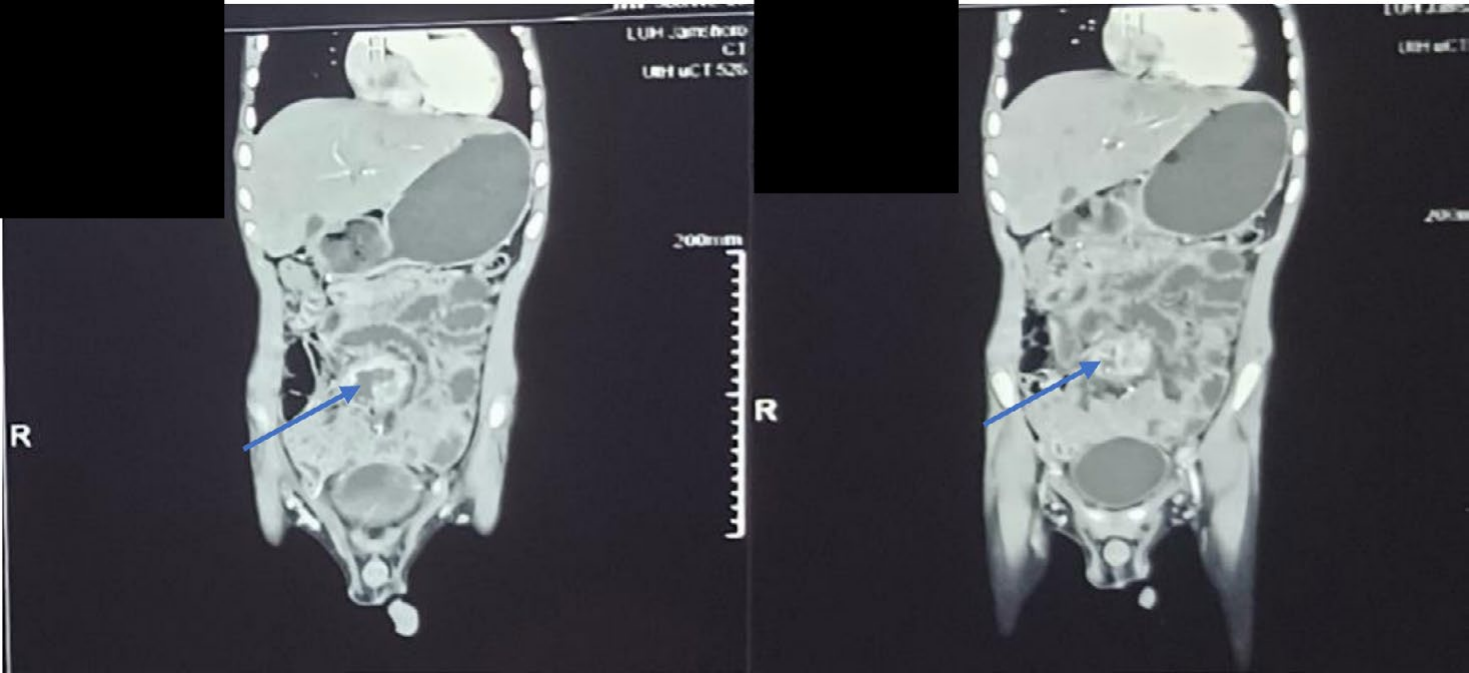
Coronal contrast‐enhanced CT scan of the abdomen demonstrating a large, complex inflammatory mass measuring 3.7 × 4.6 × 7.1 cm in the anterior abdominal wall. The mass is characterized by a thick, peripherally enhancing wall and a central necrotic component, consistent with an abscess within a urachal phlegmon (Arrow).

**FIGURE 2:**
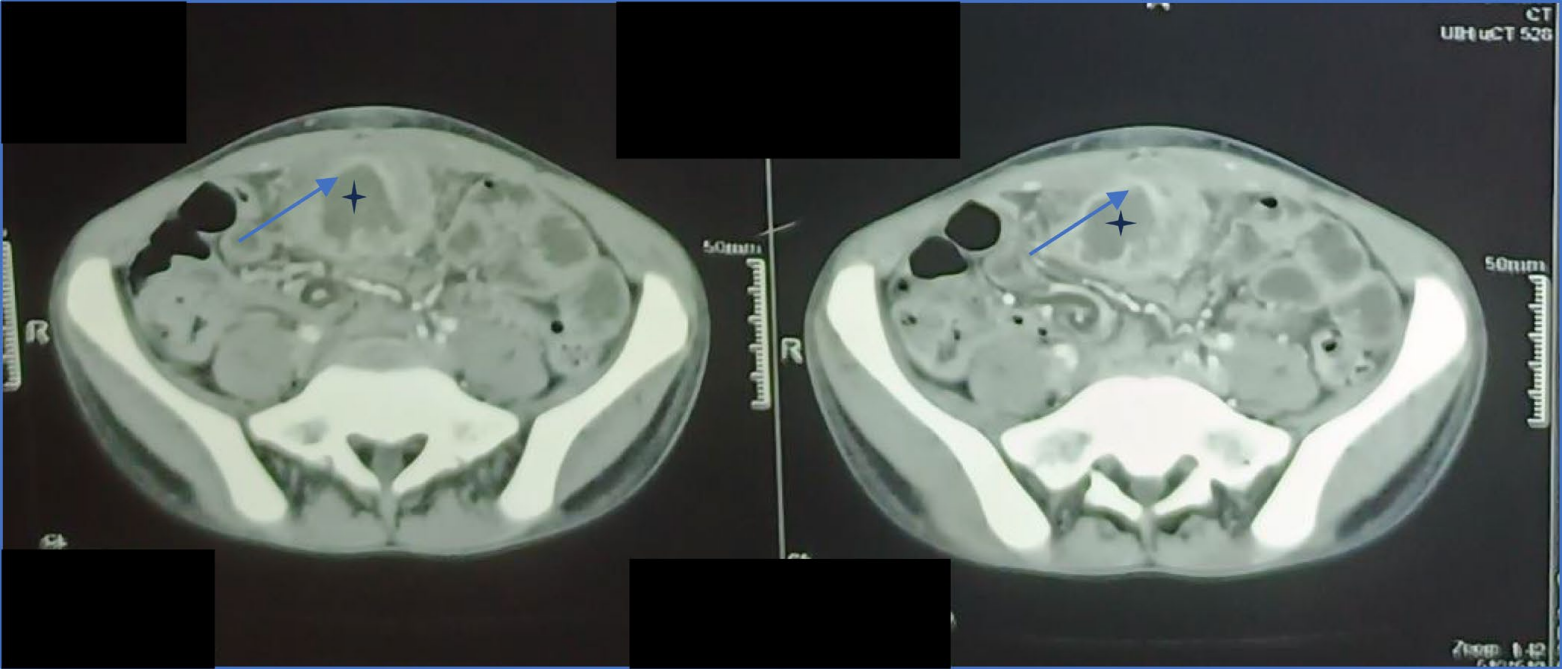
Contrast‐enhanced axial computed tomography (CT) of the abdomen. The axial view reveals a large, 7.1‐cm complex inflammatory mass (phlegmon) located in the midline of the anterior lower abdominal wall. The lesion demonstrates a thick, peripherally enhancing wall (arrow) and a central, low‐density necrotic core (asterisk). Significant inflammatory fat stranding surrounds the mass, effacing the tissue planes and confirming dense adherence to adjacent loops of the distal ileum.

Laboratory investigations revealed a significant leukocytosis of 11.9 × 10^9^/L (normal range: 4.5–11.0 × 10^9^/L) and a markedly elevated C‐reactive protein of 185 mg/L (normal range: < 5 mg/L). An urgent contrast‐enhanced computed tomography (CT) scan of the abdomen and pelvis identified a large, 3.7 × 4.6 × 7.1 cm complex inflammatory mass in the anterior abdomen inferior to the umbilicus, characterized by a thick, peripherally enhancing wall and a central necrotic core (Figures 1 and 2). Critically, there was extensive surrounding fat stranding and complete effacement of the tissue planes between the mass and adjacent ileal loops. A thin, patent tract extending from the mass inferiorly to the bladder dome confirmed its urachal origin. The primary differential diagnoses included a complicated Meckel's diverticulitis or an appendiceal abscess, but the classic midline location and connection to the bladder were highly characteristic of a complicated urachal remnant.

## 4 Treatment and Follow‐Up

**FIGURE 3:**
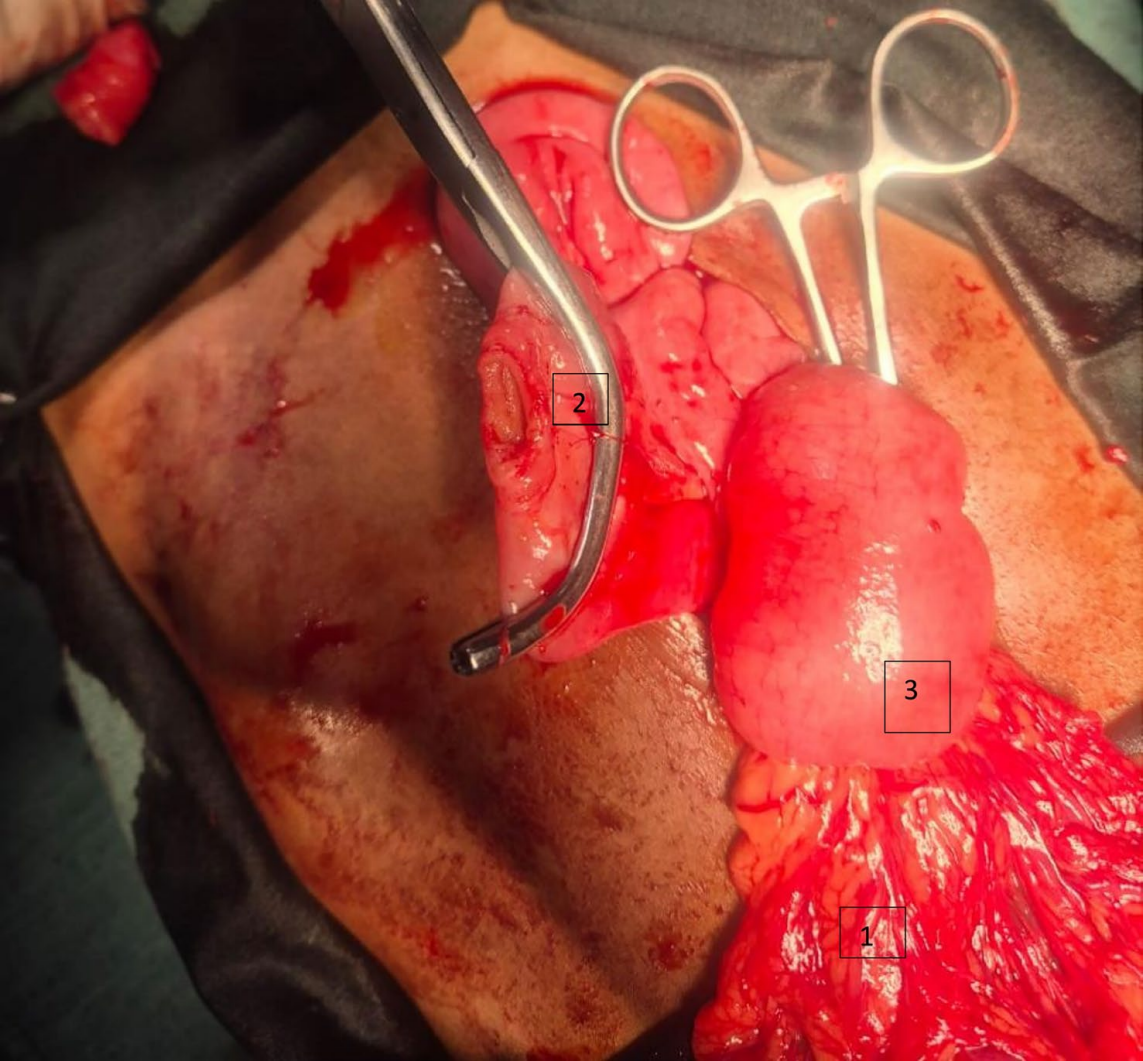
Intraoperative photograph demonstrating urachal phlegmon‐induced bowel ischemia. The large, indurated urachal mass is shown infiltrating the mesentery of the small bowel (1), leading to vascular compromise. This resulted in a dusky, nonviable 12 cm segment of the distal ileum (2), which was subsequently resected. Healthy, viable small bowel is visible proximally for comparison (3).

Following stabilization with intravenous fluids and broad‐spectrum antibiotics, the patient underwent an exploratory laparotomy. The procedure revealed a large, indurated urachal phlegmon that had entrapped and compromised the vasculature of the adjacent small bowel (Figure 3). A 12 cm segment of the distal ileum was found to be dusky, edematous, and nonviable, with clear demarcation from healthy bowel. Consequently, an en‐bloc resection of the urachal cyst, its fibrous tract to the bladder dome, and the ischemic ileal segment was performed. Bowel continuity was restored with a hand‐sewn, single‐layer end‐to‐end anastomosis.

Intraoperative cultures, obtained directly from the purulent contents of the urachal cyst, subsequently grew *Escherichia coli* . A careful intraoperative inspection confirmed that no direct communication or fistula between the cyst and the bowel was present. Histopathology of the resected specimen confirmed a benign urachal cyst with acute‐on‐chronic suppurative inflammation and transmural coagulative necrosis of the ileal segment, consistent with irreversible ischemic injury. The patient's postoperative course was unremarkable; antibiotics were tailored to culture sensitivities, and he was discharged on the fourth postoperative day. At his 2‐month follow‐up, he remained asymptomatic with no evidence of recurrence.

## 5 Discussion

This case report describes an exceptionally rare and severe complication of an infected urachal cyst in an adolescent: segmental small bowel ischemia necessitating intestinal resection. While urachal anomalies are known to present with infection [7], the progression to a dense inflammatory phlegmon causing vascular compromise is a critical finding that has been seldom reported in the pediatric literature [6].

The pathophysiology of this visceral complication is likely multifactorial, particularly given the patient's 7‐year history of an infraumbilical mass. Such a long‐standing lesion may facilitate a process of chronic, subclinical inflammation, leading to the formation of dense fibrous adhesions between the urachal remnant and adjacent bowel loops [7]. The subsequent acute infection, in this case with *E. coli* , likely triggered an aggressive inflammatory cascade within this pre‐existing fibrotic tissue, transforming the cyst into an indurated phlegmon that compromised the mesenteric vascular supply to the involved ileum. While intestinal obstruction secondary to infected urachal remnants has been documented, progression to frank vascular compromise appears to be a far more unusual event [6].

Diagnostically, this case highlights the crucial role of advanced imaging in the pediatric acute abdomen. While ultrasonography is often the first‐line modality, its utility can be limited in complex inflammatory cases [8, 9]. Contrast‐enhanced CT was invaluable here; it not only confirmed the urachal origin of the mass but, more importantly, delineated the severity of the phlegmonous process and its intimate relationship with the small bowel, findings that were critical for anticipating the need for bowel resection.

From a management perspective, the severity of this presentation informs the discussion regarding the optimal treatment of symptomatic urachal remnants. Some literature reports successful conservative management with antibiotics for uncomplicated infected cysts [7, 10, 11]. However, the development of ischemia in our patient illustrates a potential failure point of a nonoperative or delayed surgical approach in the setting of severe inflammation. Therefore, this case supports the consideration of complete surgical excision for symptomatic or chronically present urachal remnants to prevent recurrence and mitigate the risk of severe, albeit rare, complications. The choice of surgical approach must be tailored to the clinical scenario; while laparoscopic excision is increasingly utilized, the extensive inflammatory adhesions in this case necessitated an open, en‐bloc resection to ensure safe dissection [7, 12].

This report has limitations, most notably that it is a single case. The patient's unusually long 7‐year history of an untreated mass may represent a specific predisposing factor for this severe ischemic complication, and therefore, the findings may not be directly generalizable. Nevertheless, this case establishes a clear association between a long‐standing inflammatory urachal mass and the acute development of visceral ischemia, reinforcing the need for a high index of suspicion for this rare but life‐threatening event.

## 6 Conclusion

The clinical course of this patient demonstrates that a long‐standing, infected urachal cyst has the potential to evolve into an aggressive phlegmonous mass capable of causing visceral ischemia. While this represents a rare event, it is a life‐threatening complication that highlights the fact that symptomatic urachal remnants are not uniformly benign. Therefore, in patients presenting with an acute abdomen and an inflammatory infraumbilical mass, a complicated urachal anomaly should be considered in the differential diagnosis, and the possibility of vascular compromise should be investigated if signs of severe inflammation are present.

## Author Contributions

**Sheeraz Ali:** data curation, formal analysis, investigation, methodology, project administration, writing – original draft, writing – review and editing. **FNU Saliha:** conceptualization, data curation, formal analysis, methodology, project administration, writing – original draft, writing – review and editing. **Bilal Ahmad:** formal analysis, investigation, methodology, supervision, writing – original draft, writing – review and editing. **Abdul Eizad Asif:** methodology, project administration, validation, visualization, writing – original draft, writing – review and editing. **Khalil El Abdi:** conceptualization, data curation, methodology, project administration, writing – original draft, writing – review and editing. **Bilal Aslam:** conceptualization, data curation, methodology, project administration, writing – original draft, writing – review and editing. **Fazeela Bibi:** conceptualization, methodology, project administration, writing – original draft, writing – review and editing. **Said Hamid Sadat:** conceptualization, data curation, formal analysis, writing – original draft, writing – review and editing.

## Funding

The authors have nothing to report.

## Consent

Informed written consent was obtained from the patient.

## Conflicts of Interest

The authors declare no conflicts of interest.

## Data Availability

Data available on request from the authors.
